# Antimicrobial Activity of Peptide-Coupled Antisense Peptide Nucleic Acids in Streptococcus pneumoniae

**DOI:** 10.1128/spectrum.00497-22

**Published:** 2022-11-02

**Authors:** Gina Barkowsky, Corina Abt, Irina Pöhner, Adam Bieda, Sven Hammerschmidt, Anette Jacob, Bernd Kreikemeyer, Nadja Patenge

**Affiliations:** a Institute of Medical Microbiology, Virology and Hygiene, University Medicine Rostock, Rostock, Germany; b Department of Molecular Genetics and Infection Biology, Interfaculty Institute for Genetics and Functional Genomics, Center for Functional Genomics of Microbes, University of Greifswald, Greifswald, Germany; c Peps4LS GmbH, Heidelberg, Germany; d Functional Genome Analysis, Deutsches Krebsforschungszentrum, Heidelberg, Germany; Indian Institute of Science Bangalore

**Keywords:** antimicrobial activity, antimicrobial therapy, antisense molecules, pneumococcus, *Streptococcus pneumoniae*, peptide nucleic acid, cell penetrating peptide

## Abstract

Streptococcus pneumoniae is the most common cause of community-acquired pneumonia and is responsible for multiple other infectious diseases, such as meningitis and otitis media, in children. Resistance to penicillins, macrolides, and fluoroquinolones is increasing and, since the introduction of pneumococcal conjugate vaccines (PCVs), vaccine serotypes have been replaced by non-vaccine serotypes. Antisense peptide nucleic acids (PNAs) have been shown to reduce the growth of several pathogenic bacteria in various infection models. PNAs are frequently coupled to cell-penetrating peptides (CPPs) to improve spontaneous cellular PNA uptake. In this study, different CPPs were investigated for their capability to support translocation of antisense PNAs into S. pneumoniae. HIV-1 TAT- and (RXR)_4_XB-coupled antisense PNAs efficiently reduced the viability of S. pneumoniae strains TIGR4 and D39 *in vitro*. Two essential genes, *gyrA* and *rpoB*, were used as targets for antisense PNAs. Overall, the antimicrobial activity of anti-*gyrA* PNAs was higher than that of anti-*rpoB* PNAs. Target gene transcription levels in S. pneumoniae were reduced following antisense PNA treatment. The effect of HIV-1 TAT- and (RXR)_4_XB-anti-*gyrA* PNAs on pneumococcal survival was also studied *in vivo* using an insect infection model. Treatment increased the survival of infected Galleria mellonella larvae. Our results represent a proof of principle and may provide a basis for the development of efficient antisense molecules for treatment of S. pneumoniae infections.

**IMPORTANCE** Streptococcus pneumoniae is the most common cause of community-acquired pneumonia and is responsible for the deaths of up to 2 million children each year. Antibiotic resistance and strain replacement by non-vaccine serotypes are growing problems. For this reason, S. pneumoniae has been added to the WHO “global priority list” of antibiotic-resistant bacteria for which novel antimicrobials are most urgently needed. In this study, we investigated whether CPP-coupled antisense PNAs show antibacterial activity in S. pneumoniae. We demonstrated that HIV-1 TAT- and (RXR)4XB-coupled antisense PNAs were able to kill S. pneumoniae *in vitro*. The specificity of the antimicrobial effect was verified by reduced target gene transcription levels in S. pneumoniae. Moreover, CPP-antisense PNA treatment increased the survival rate of infected Galleria mellonella larvae *in vivo*. Based on these results, we believe that efficient antisense PNAs can be developed for the treatment of S. pneumoniae infections.

## INTRODUCTION

Streptococcus pneumoniae (pneumococcus) are Gram-positive pathogens which colonize the upper respiratory tract and cause lower respiratory tract infections, otitis media in children, conjunctivitis, bacteremia, and meningitis (1). Pneumococci are the main cause of community-acquired pneumonia (CAP), which mostly affects children under the age of five, the elderly, and patients with underlying comorbidities (2, 3). Infections occur more frequently during the cold season and in conjunction with viral infections of the upper respiratory tract (4, 5). The global burden of pneumococcal disease is high, being responsible for the deaths of up to 2 million children each year, most of whom live in developing countries (6). Pneumococcal pneumonia can be prevented by immunization and adequate nutrition, and by addressing environmental factors.

The introduction of pneumococcal conjugate vaccines (PCV) reduced invasive pneumococcal disease and carriage rates in immunized children, leading to protection of the unimmunized population, also referred to as herd immunity (7, 8). Globally, pneumococcal deaths in children declined by approximately 51% from 2000 to 2015 (9). Over the years, serotype replacement has resulted in the rising prevalence of non-vaccine serotypes in carriage and disease, limiting the protective effect of PCV (10). The genetic adaptability of S. pneumoniae facilitates the generation of recombinants which adjust to the selective pressures exerted by vaccines and antimicrobials, leading to the spread of antibiotic resistance and evasion of vaccine-induced immunity (11, 12). Consequently, S. pneumoniae is included in the WHO’s “global priority list” (priority 3) of antibiotic-resistant bacteria for which novel antimicrobials are most urgently needed (13). To limit the use of broad-spectrum antibiotics, the development of species-specific antimicrobials is desirable (14). Antisense oligonucleotides (ASOs) targeting the start codon region of mRNAs have the potential to act specifically on the species level (15). Inhibiting the initiation of the translation of essential genes or virulence factors affects bacterial survival or pathogenesis, respectively.

Peptide nucleic acids (PNAs) are synthetic DNA analogues featuring a pseudopeptide backbone to which nucleobases are bound. PNAs can hybridize to DNA and RNA with high affinity (16). Their chemical composition, together with their resistance to proteases and nucleases, renders PNAs very stable (17). These properties make them interesting tools for gene silencing. However, surface barriers, including membranes, cell walls, and capsules, restrict bacterial uptake. Translocation of PNAs into cells can be facilitated by coupling them to cell-penetrating peptides (CPPs), which have been widely studied for cargo delivery into eukaryotic cells (18).

The efficiency of CPP-PNA uptake into bacteria depends on the species and can even vary between strains and serotypes depending on different surface properties. The antimicrobial effects of CPP-antisense PNAs have been intensively studied in Gram-negative bacteria (19). So far, a limited number of CPPs have identified which effectively support PNA uptake into Gram-positive bacteria, including Listeria monocytogenes and Staphylococcus aureus (20, 21). In a recent study, we systematically screened for CPPs which facilitate PNA translocation into Streptococcus pyogenes. We identified three CPPs which were able to support antimicrobial activity of anti-*gyrA* PNAs: HIV-1 TAT, K8, and (RXR)_4_XB (22). Here, we investigated the effects of HIV-1 TAT, K8, and (RXR)_4_XB on antisense PNA activity in S. pneumoniae.

## RESULTS

### Design of CPP-coupled antisense PNAs specific for *S. pneumoniae*.

In a previous study, we observed the antimicrobial effects of peptide-coupled anti-*gyrA* antisense PNAs specific for S. pyogenes (22). Because carrier molecules influence cargo uptake in a species-specific manner, we wanted to identify CPPs which mediated the uptake of antisense PNAs into S. pneumoniae. As antisense targets, we selected the essential genes *gyrA* and *rpoB*, which encode a subunit of the DNA gyrase and the bacterial RNA polymerase, respectively. These two enzymes have been used as antimicrobial targets in several studies, including antisense PNA experiments in Gram-positive pathogens (22–24). Peptides were coupled to PNAs via a flexible ethylene glycol linker (8-amino-3, 6-dioxaoctanoic acid). The sequences of all peptide-conjugated PNAs used in this study are listed in Table 1. Scrambled control PNAs (scrambled PNAs, scPNAs) were composed of the same base pairs as the antisense PNAs but featured a randomized order.

**TABLE 1:** CPP-PNA conjugates for antisense-studies in Streptococcus pneumoniae^*a*^

CPP	CPP-PNA designation	CPP-PNA sequence	Reference
HIV-1 TAT (48–57)	TAT-anti-*gyrA* PNA	GRKKRRQRRRYK-eg-tgcattaata	47
	TAT-anti-*gyrA* scPNA	GRKKRRQRRRYK-eg-aatgattact	
	TAT-anti-*rpoB* PNA	GRKKRRQRRRYK-eg-ctgccaagatga	
	TAT-anti-*rpoB* scPNA	GRKKRRQRRRYK-eg-cgtagtccaaag	
Oligolysin (K8)	K8-anti-*gyrA* PNA	KKKKKKKK-eg-tgcattaata	31
	K8-anti-*gyrA* scPNA	KKKKKKKK-eg-aatgattact	
(RXR)_4_XB	(RXR)_4_XB-anti-*gyrA* PNA	RXRRXRRXRRXRXB-eg-tgcattaata	48
	(RXR)_4_XB-anti-*gyrA* scPNA	RXRRXRRXRRXRXB-eg-aatgattact	
	(RXR)_4_XB-anti-*rpoB* PNA	RXRRXRRXRRXRXB-eg-ctgccaagatga	
	(RXR)_4_XB-anti-*rpoB* scPNA	RXRRXRRXRRXRXB-eg-cgtagtccaaag	

a CPP, cell-penetrating peptide; PNA, peptide nucleic acid; eg, 8-amino-3,6-dioxaoctan acid; X, 6-aminohexanoic acid; B, β-alanine.

### Antimicrobial effects of CPP-coupled antisense PNAs on *S. pneumoniae*.

**FIG 1:**
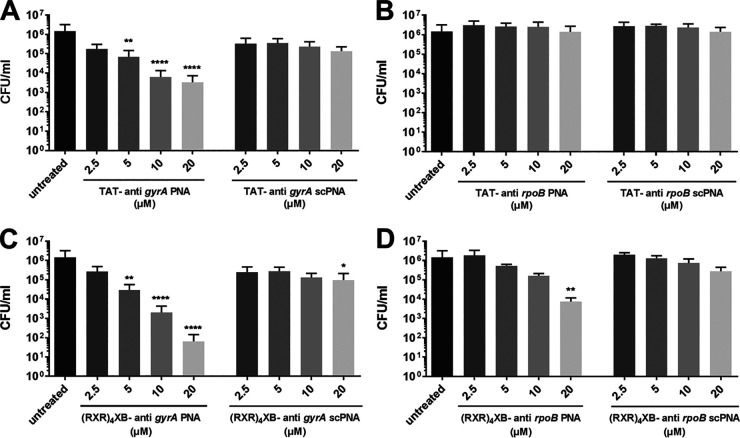
Concentration-dependent reduction of the pneumococcal CFU/mL following treatment of Streptococcus pneumoniae TIGR4 with cell-penetrating peptide (CPP)-antisense peptide nucleic acids (PNAs) for 6 h. scPNA, scrambled PNA controls. (A) Treatment with TAT-anti-*gyrA* PNAs. (B) Treatment with TAT-anti-*rpoB* PNAs. (C) Treatment with (RXR)_4_XB-anti-*gyrA* PNAs. (D) Treatment with (RXR)_4_XB-anti-*rpoB* PNAs. Data are presented as means and standard deviation. Statistical significance was determined by one-way analysis of variance (ANOVA) with multiple comparisons. Differences between PNA conjugate samples and mock control (untreated) are shown as *, *P* ≤ 0.05; **, *P* ≤ 0.01; and ****, *P* ≤ 0.0001. Sample size: *n* = 5.

The impact of different CPPs on the efficacy of antisense PNAs targeting *gyrA* and *rpoB* in S. pneumoniae was tested using an *in vitro* killing assay (25). S. pneumoniae strains were incubated for 6 h with CPP-antisense PNA conjugates. We determined the reduction of bacterial CFU/mL caused by different CPP-coupled antisense PNA constructs compared to an untreated control. Three different CPPs were tested: HIV-1 TAT (TAT), oligolysin (K8), and (RXR)_4_XB (Table 1). Concentration-dependent bactericidal activity was investigated by treatment of S. pneumoniae TIGR4 in a CPP-antisense PNA conjugate concentration range of 2.5 to 20 μM (Fig. 1).

The CFU in treated samples was significantly reduced compared to the untreated control sample when TAT-anti-*gyrA* PNA was applied at a concentration range of 5 to 20 μM (Fig. 1A). A 50% inhibitory concentration (IC_50_) of 0.4 μM was determined for TAT-anti-*gyrA* PNA. No reduction of bacterial counts was observed following incubation of S. pneumoniae TIGR4 with TAT-anti-*rpoB* PNA (Fig. 1B). TAT-conjugated scPNAs caused no significant reduction of CFU/mL following treatment (Fig. 1A/B), indicating a sequence-specific bactericidal effect of the TAT-anti-*gyrA* PNA construct. (RXR)_4_XB-anti-*gyrA* PNA and (RXR)_4_XB-anti-*rpoB* PNA caused significant decreases in S. pneumoniae TIGR4 bacterial counts following treatment (Fig. 1C/D).

In contrast, no reduction of CFU/mL was observed following incubation with K8-conjugated anti-*gyrA* PNA (data not shown). (RXR)_4_XB-anti-*gyrA* was the most efficient construct, leading to a log CFU reduction of 4 following treatment with a concentration of 20 μM CPP-PNA. The IC_50_ of (RXR)_4_XB-anti-*gyrA* PNA was 0.63 μM. (RXR)_4_XB-conjugated scPNAs caused a slight reduction in CFU/mL following treatment (log CFU reduction ≤1) (Fig. 1C and D). Considering the toxic effects of CPPs, we calculated the specific antimicrobial activity of the CPP-antisense PNAs by subtracting the log CFU reduction caused by CPP-scrambled control PNAs from the log CFU reduction caused by the corresponding CPP-antisense PNA (Table 2). In S. pneumoniae TIGR4, specific activities of log CFU reduction ≥1 were observed following treatment with 10 μM (RXR)_4_XB-anti-*gyrA* PNA and TAT-anti-*gyrA* PNA, and with 20 μM (RXR)_4_XB-anti-*rpoB* PNA. No specific activity could be detected using TAT-anti-*rpoB* PNA (Table 2).

**TABLE 2:** Specific antimicrobial activity of CPP-antisense PNA conjugates in Streptococcus pneumoniae^*a*^

CPP-PNA	C_PNA_ (μM)	Δlog CFU reduction^*b*^ in S. pneumoniae strains		
		TIGR4	D39	19F
(RXR)_4_XB-anti-*gyrA*	2.5	0	0	0.72
	5	0.98	0	1.20
	10	1.80	0.42	1.21
	20	3.18	2.23	1.48
TAT-anti-*gyrA*	2.5	0.27	0	0.50
	5	0.70	0	0.51
	10	1.57	1.30	0.22
	20	1.60	1.96	0.32
(RXR)_4_XB-anti-*rpoB*	2.5	0	0	0.18
	5	0.40	0.16	0.17
	10	0.66	0.61	0.16
	20	1.58	1.98	0.56
TAT-anti-*rpoB*	2.5	0	0	0.05
	5	0	0.11	0.05
	10	0	0.16	0
	20	0	0.51	0

a CPP, cell-penetrating peptide; PNA, peptide nucleic acid.

b Log CFU reduction CPP-antisense PNA – log CFU reduction CPP-scPNA.

Similar results were obtained with S. pneumoniae strain D39 (Fig. S1). (RXR)_4_XB-coupled antisense PNAs were more efficient than TAT-coupled antisense PNAs. K8-antisense PNAs did not show any antimicrobial effect (data not shown). The difference between the target genes anti-*gyrA* and anti-*rpoB* was less pronounced in D39 than in TIGR4 (Table 2). No significant reduction of bacterial counts was observed following incubation with CPP-conjugated scPNAs (Fig. S1).

Compared to S. pneumoniae strains TIGR4 and D39, strain 19F was clearly affected by incubation with CPP-coupled scPNAs (Fig. S2). Specific antimicrobial activity of TAT-antisense PNAs was low, regardless of the target gene (Fig. S2, Table 2).

Treatment with (RXR)_4_XB-anti-*gyrA* PNA caused a relevant reduction in S. pneumoniae strain 19F CFU. Treatment with 10 μM (RXR)_4_XB-anti-*gyrA* PNA exhibited a specific log CFU reduction of ≥1 (Fig. S2C and D, Table 2). Overall, (RXR)_4_XB-anti-*gyrA* PNA had the highest antimicrobial activity of all constructs tested. It achieved a specific log CFU reduction of >1 in all S. pneumoniae strains investigated in this study (Table 2).

CPP-coupled scrambled PNAs were used as controls throughout this study because the cytotoxicity of CPPs is highly dependent on the cargo used (26). Accordingly, the antibacterial effects of (RXR)_4_XB and TAT peptides on S. pneumoniae TIGR4 were higher than those of the conjugated peptides (Fig. S3).

### Bactericidal kinetics of CPP-coupled antisense PNAs in *S. pneumoniae*.

**FIG 2:**
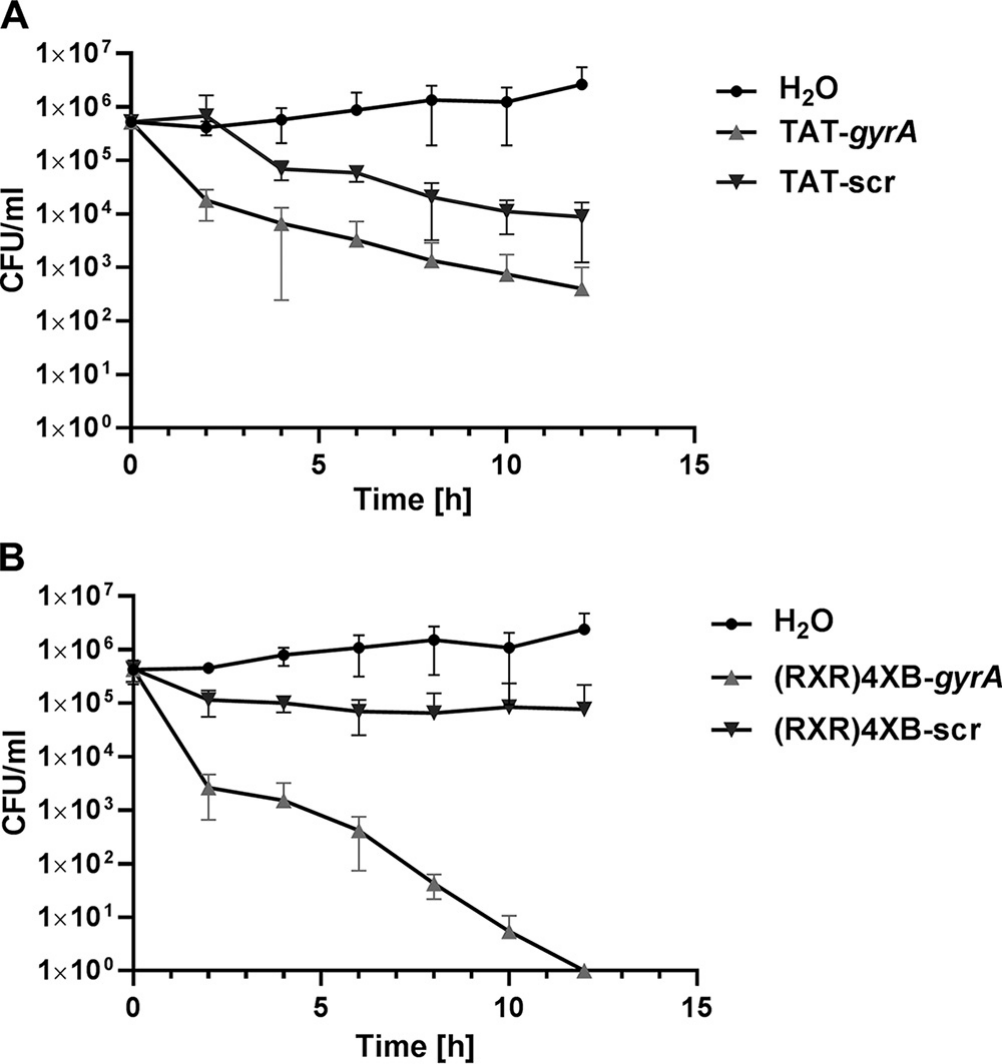
Killing kinetics of CPP-anti-*gyrA* PNA treatment in S. pneumoniae *strain* TIGR4. (A) Bacterial counts following treatment with 10 μM TAT-anti-*gyrA* PNA or 10 μM TAT-anti-*gyrA* scPNAs. (B) Bacterial counts following treatment with 10 μM (RXR)_4_XB-anti-*gyrA* PNAs or 10 μM (RXR)_4_XB-anti-*gyrA* scPNAs. Data are presented as means and standard deviation. Sample size: *n* = 3.

The antimicrobial activity kinetics of TAT- and (RXR)_4_XB-conjugated anti-*gyrA* PNA constructs in S. pneumoniae TIGR4 were studied using an *in vitro* time-killing assay. Bacteria were treated with 10 μM CPP-antisense PNAs or scrambled control PNAs (Fig. 2). Samples were collected every 2 h following antisense treatment. CFU/mL was determined by plating of serial dilutions and plotted over time. Treatment with TAT-anti-*gyrA* PNA led to a steady reduction in bacterial counts, but no clearance was achieved over the course of the experiment (Fig. 2A). Following treatment with TAT-conjugated scPNA, reduced bacterial counts were observed 4 h post-treatment and continued throughout the experiment, indicating an unspecific toxic effect of the CPP. In contrast, (RXR)_4_XB-conjugated anti-*gyrA* PNA treatment led to a continuous reduction in S. pneumoniae CFU until complete eradication after 12 h. (RXR)_4_XB-conjugated scPNA did not cause reduced bacterial counts under these conditions.

### CPP-coupled antisense PNAs affect the abundance of target gene transcripts in *S. pneumoniae*.

**FIG 3:**
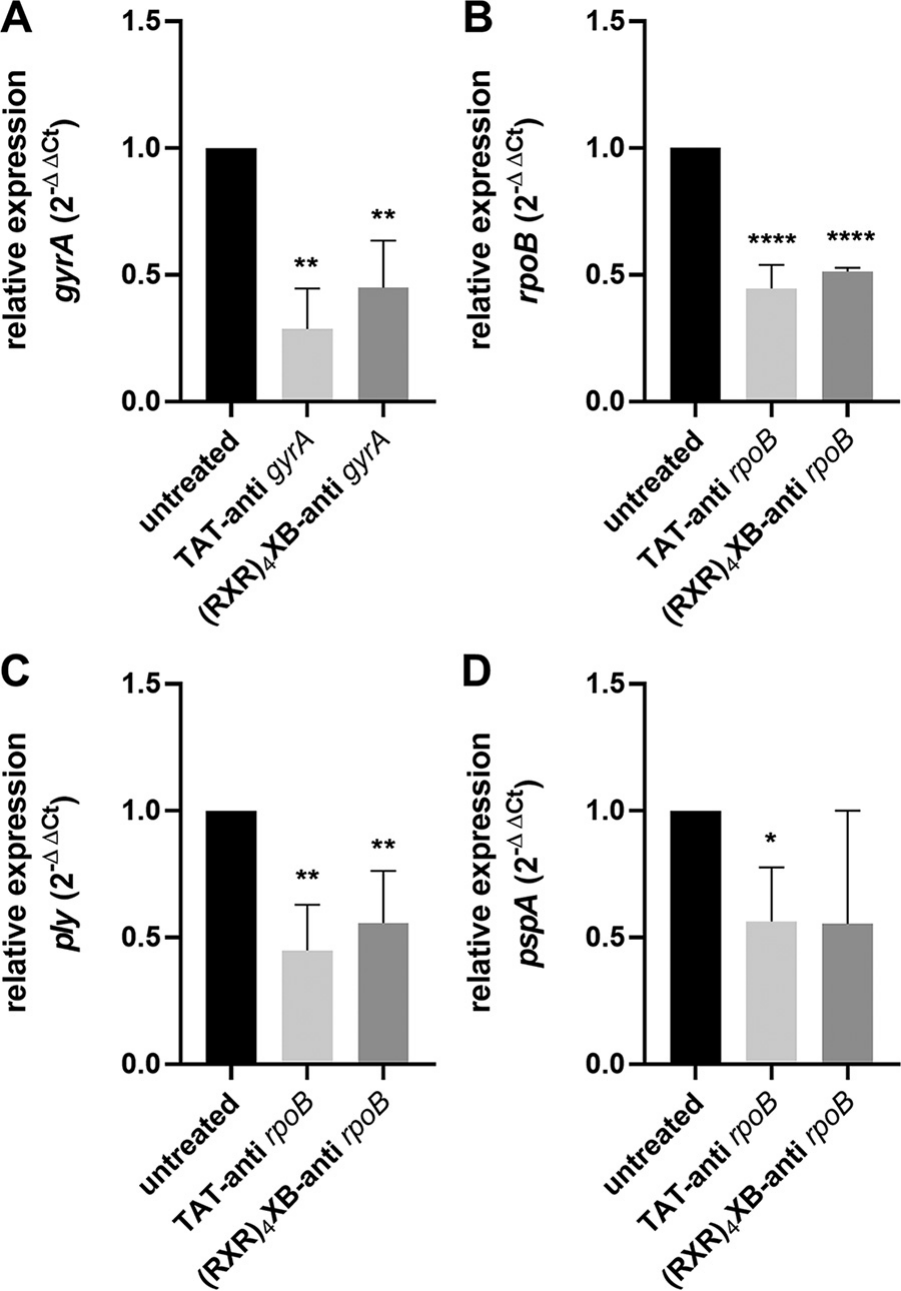
Relative expression of target genes and virulence genes in S. pneumoniae *strain* TIGR4 following treatment with CPP-antisense PNAs. The 5S rRNA gene served as internal control. Relative expression was calculated using the threshold cycle (2^–ΔΔ^*^CT^*) method. (A) Treatment with 7.5 μM TAT-anti-*gyrA* PNAs and 7.5 μM (RXR)_4_XB-anti-*gyrA* PNAs. Relative expression of *gyrA*. (B) Treatment with 10 μM TAT-anti-*rpoB* PNAs and 10 μM (RXR)_4_XB-anti-*rpoB* PNAs. Relative expression of *rpoB*. (C) Treatment with 10 μM TAT-anti-*rpoB* PNAs and 10 μM (RXR)_4_XB-anti-*rpoB* PNAs. Relative expression of *ply*. (D) Treatment with 10 μM TAT-anti-*rpoB* PNAs and 10 μM (RXR)_4_XB-anti-*rpoB* PNAs. Relative expression of *pspA*. Data are presented as means and standard deviation. Statistical significance was determined by one-way ANOVA with multiple comparisons. Differences between PNA conjugate samples and the mock control (untreated) are shown as *, *P* ≤ 0.05; **, *P* ≤ 0.01; and ****, *P* ≤ 0.0001. Sample size: *n* = 3.

We investigated the influence of treatment with CPP-conjugated antisense PNAs on the amount of target gene mRNA in S. pneumoniae using reverse transcription-quantitative PCR (RT-qPCR) (Fig. 3). S. pneumoniae strain TIGR4 was treated with a sublethal dose (log CFU reduction of <2) of CPP-PNA conjugates. Transcript abundance of the 5S rRNA gene was used for normalization (27). Target gene mRNA levels in mock-treated bacteria served as controls. Treatment with 7.5 μM CPP-anti-*gyrA* PNAs and 10 μM CPP-anti-*rpoB* PNAs led to significant reductions in the respective target gene transcripts compared to the untreated control sample (Fig. 3).

Following treatment with TAT-anti-*gyrA* PNA and (RXR)_4_XB-anti-*gyrA* PNA, *gyrA* mRNA levels decreased to 29% and 45%, respectively (Fig. 3A). Transcript levels of *rpoB* were reduced to 45% and 51%, respectively, upon incubation with TAT-anti-*rpoB* PNA and (RXR)_4_XB-anti-*rpoB* PNA (Fig. 3B). The addition of higher concentrations of anti-*rpoB* PNAs was required to obtain a significant reduction in relative target gene expression. These results underline the higher antimicrobial efficiency of anti-*gyrA* PNAs we observed in S. pneumoniae TIGR4 (Fig. 1).

### Antisense PNA-mediated *rpoB* downregulation affects transcription of virulence genes.

Because the product of *rpoB* is the β-subunit of the bacterial RNA-polymerase, which is responsible for RNA synthesis, downregulation of *rpoB* by treatment with antisense PNAs is expected to affect gene expression. Therefore, we tested the relative expression of two virulence factor genes following treatment with 10 μM CPP-anti-*rpoB* PNAs compared to expression in the mock-treated control as an example (Fig. 3C and D).

Following TAT-anti-*rpoB* PNA treatment, the expression of *ply*, which codes for pneumolysin, was reduced to 45%. Treatment with (RXR)_4_XB-anti-*rpoB* PNA reduced *ply* expression to 56% (Fig. 3C). Following treatment with TAT-anti-*rpoB* PNA, the expression of *pspA*, which codes for pneumococcal surface protein A, was reduced to 57%. In contrast, there was high variability in relative *pspA* transcription levels following treatment with (RXR)_4_XB-anti-*rpoB* PNA, and no significant reduction could be observed (Fig. 3D).

### Antimicrobial effects of CPP-antisense PNA conjugates in a cell-based infection model.

**FIG 4:**
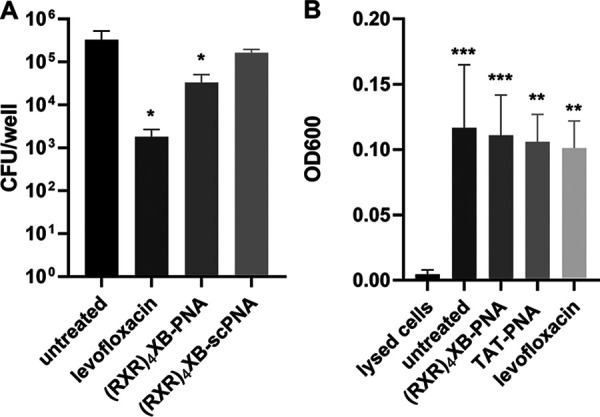
Antimicrobial and cytotoxic effects of (RXR)4XB-anti-*gyrA* PNA in a cell-based infection system. (A) Detroit 562 cells infected with S. pneumoniae TIGR4 and subsequently treated with 1 μg levofloxacin, 20 μM (RXR)_4_XB-anti-*gyrA* PNA, or 20 μM (RXR)_4_XB-anti-*gyrA* scPNA, respectively. Sample size: *n* = 3. Statistical significance was determined by one-way ANOVA with multiple comparisons. Differences between PNA conjugate samples and mock control (untreated) are shown as *, *P* ≤ 0.05. (B) Detroit 562 cells were lysed and treated with 20 μM (RXR)_4_XB-PNA or 20 μM TAT-PNA, respectively. Biomass was stained with crystal violet, bound crystal violet was extracted, and the optical density of the solution was measured at 600 nm. Sample size: *n* = 4. Statistical significance was determined by one-way ANOVA with multiple comparisons. Differences between samples and maximal cell lysis are shown as **, *P* ≤ 0.01; ***, *P* ≤ 0.001.

Detroit 562 human pharyngeal epithelial cells were infected with S. pneumoniae TIGR4 at a multiplicity of infection (MOI) of 5. Cells were treated with 20 μM (RXR)_4_XB-anti-*gyrA* PNA or scrambled control PNA, respectively. As a positive control, 1 μg levofloxacin, corresponding to 3× MIC, was added. Following incubation for 3 h at 37°C, bacterial CFU counts were determined for CPP-antisense PNA-treated samples and compared to those in nontreated samples and in samples containing scrambled control PNAs (Fig. 4A). Treatment with (RXR)_4_XB-anti-*gyrA* caused a log CFU reduction of 1. Addition of levofloxacin led to a log CFU reduction of 2. No significant changes in bacterial CFU were observed following application of (RXR)_4_XB-conjugated scPNAs in comparison to the untreated control.

We investigated the potential cytotoxic effects of CPP-PNAs on Detroit 562 cells by measuring cellular viability using a crystal violet assay (Fig. 4B). Cells were incubated with 20 μM (RXR)_4_XB-PNA or TAT-PNA, respectively. Untreated cells or cells treated with levofloxacin served as negative controls. Cell lysis was used as positive control for cell death. No cell death was observed following treatment with levofloxacin or CPP-PNAs. Similar results were observed using a lactate dehydrogenase (LDH) leakage assay. LDH release after treatment of Detroit 562 cells was compared to maximum LDH release (Fig. S4). Application of 20 μM (RXR)_4_XB-PNA, 20 μM TAT-PNA, or 1 μg levofloxacin caused 5%, 6%, or 3% of maximum LDH release, respectively. Together, these results indicate that CPP-PNA conjugates have low cytotoxic activity comparable to that of the conventional antibiotic levofloxacin.

### Evaluation of CPP-antisense PNA conjugates in a *G. mellonella* infection model.

Antimicrobial efficiency of CPP-antisense PNA conjugates was evaluated *in vivo* employing a G. mellonella infection and treatment model (22, 28). Larvae were infected with S. pneumoniae strain TIGR4 and subsequently treated with 10 nmol CPP-anti-*gyrA* PNAs. Treatment with levofloxacin served as a positive control. Larval survival was observed over 7 days.

**FIG 5:**
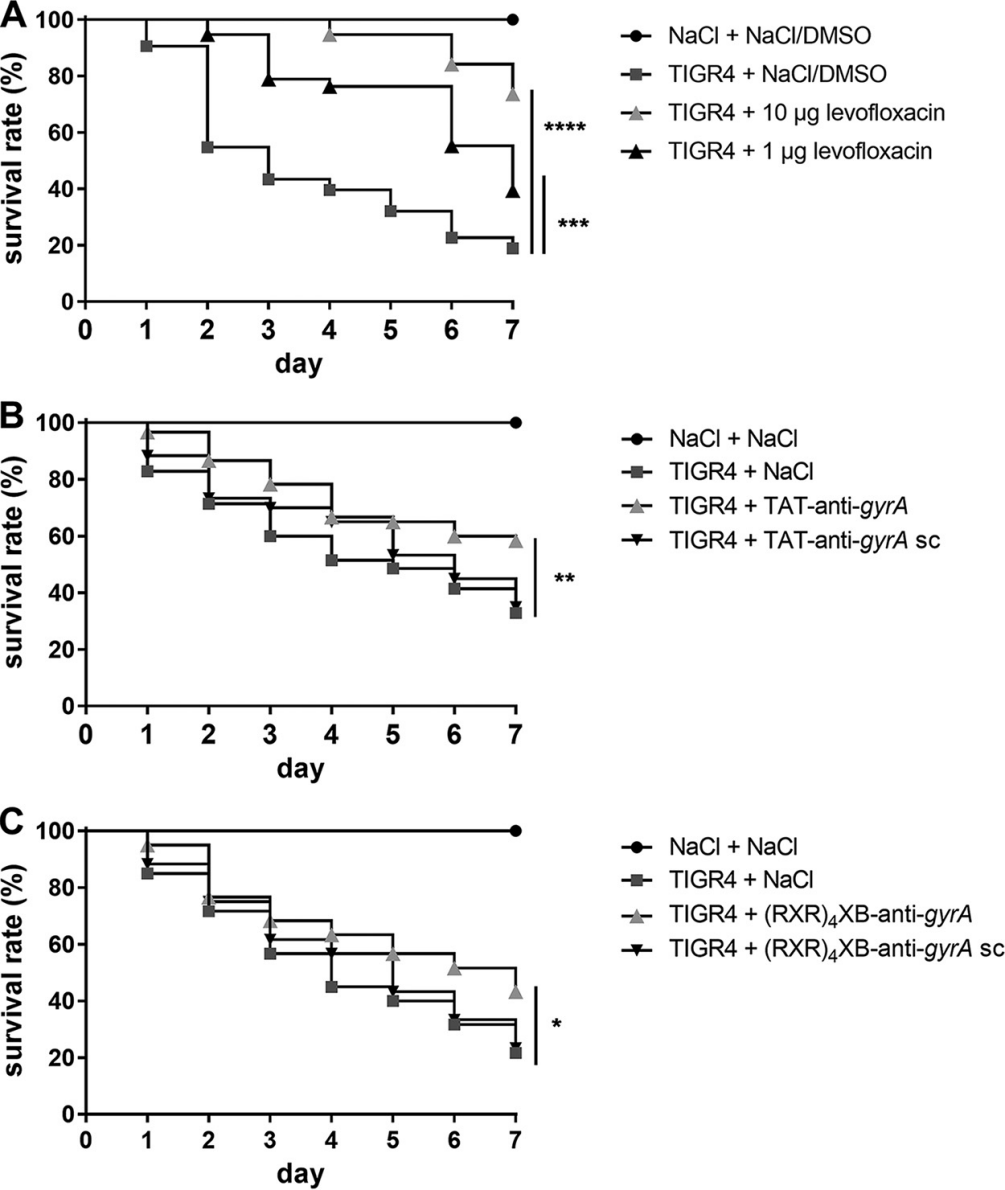
Survival of Galleria mellonella larvae treated with 10 nmol CPP-PNAs following infection with S. pneumoniae TIGR4. (A) G. mellonella larvae infected with S. pneumoniae TIGR4 and subsequently treated with 10 or 1 μg levofloxacin. Sample size: *n* = 40 larvae per group. (B) G. mellonella larvae infected with S. pneumoniae TIGR4 and subsequently treated with TAT-anti-*gyrA* PNA or TAT-anti-*gyrA* scPNA, respectively. Sample size: *n* = 60 larvae per group. (C) G. mellonella larvae infected with S. pneumoniae TIGR4 and subsequently treated with (RXR)_4_XB-anti-*gyrA* PNA or (RXR)_4_XB-anti-*gyrA* scPNA, respectively. Sample size: *n* = 60 larvae per group. Statistical significance was determined using the log-rank test. Differences between curves are shown as *, *P* ≤ 0.05; **, *P* ≤ 0.01; ***, *P* ≤ 0.001; and ****, *P* ≤ 0.0001.

The administration of 10 or 1 μg levofloxacin, corresponding to approximately 25× or 2.5× MIC, respectively, increased the survival of infected larvae from 19% to 74% or 40%, respectively (Fig. 5A). Larvae treated with TAT-anti-*gyrA* PNA or (RXR)_4_XB-anti-*gyrA* PNA exhibited increased survival compared to mock-treated larvae or those treated with CPP-scPNA (Fig. 5B/C). The survival rate was 25% or 22% higher following injection of TAT-anti-*gyrA* PNA or (RXR)_4_XB-anti-*gyrA* PNA, respectively, which is comparable to the effect of 1 μg levofloxacin (21%). Treatment of S. pneumoniae strain TIGR4-infected larvae with TAT-anti-*rpoB* PNA or (RXR)_4_XB-anti-*rpoB* PNA did not increase survival under these conditions (data not shown). These results are in accordance with the lower antimicrobial activity of these constructs compared to that of the anti-*gyrA* molecules we observed *in vitro* (Fig. 1).

**FIG 6:**
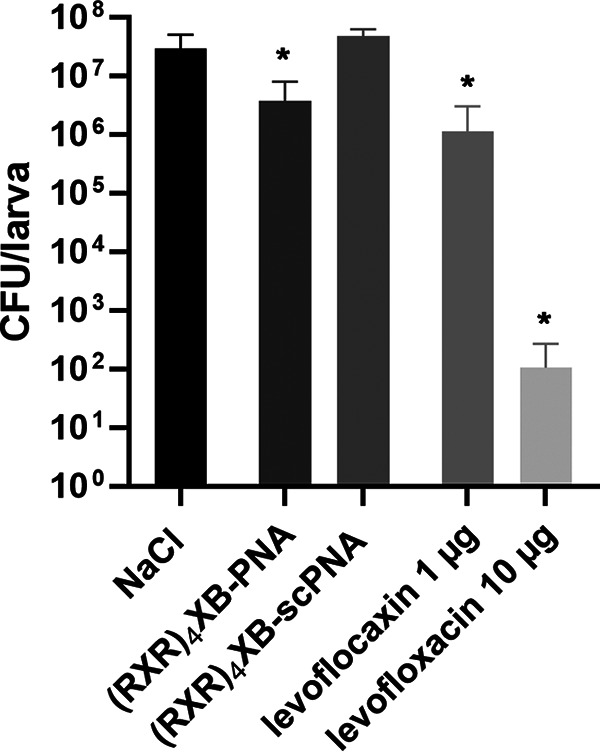
Bacterial load of G. mellonella larvae following infection with S. pneumoniae TIGR4. G. mellonella larvae infected with S. pneumoniae TIGR4 and subsequently treated with either levofloxacin or (RXR)_4_XB-PNAs. After 24 h, larvae were homogenized. Bacterial load was determined by serial dilution, plating on selective medium, and CFU calculation. Sample size: *n* = 4 larvae per group in three independent experiments. Data are presented as means and standard deviation. Statistical significance was determined by one-way ANOVA with multiple comparisons. Differences between treated samples and mock control (NaCl) are shown as *, *P* ≤ 0.05.

To establish the antibacterial effect of PNAs in the invertebrate infection model, we assessed the bacterial loads in infected larvae (Fig. 6). Larvae were infected and subsequently treated with NaCl (mock), 10 nmol CPP-PNA, or levofloxacin (positive control), respectively. From each group, four larvae were homogenized at 24 h postinfection. CFU/larva were determined following serial dilutions of the suspension and plating on selective medium. Treatment with (RXR)_4_XB-anti-*gyrA* caused a log CFU reduction of 1 compared to that in the mock-treated larvae. The addition of 1 or 10 μg levofloxacin led to a log CFU reduction of 2 or 6, respectively. In contrast, treatment with (RXR)_4_XB-conjugated scPNAs caused no significant changes in the bacterial CFU in comparison to the untreated control, suggesting a target gene-specific antimicrobial effect in the infected larvae.

## DISCUSSION

Antisense molecules are potential alternative anti-infective therapeutics which provide several advantages compared to conventional antibiotics. They combine a high specificity toward their target structures with broad application versatility. Furthermore, there are no preexisting natural resistance mechanisms. It is expected that upon application of antisense therapeutics, genetic resistance will be developed. In this case, it is easy to respond in a timely manner by adjusting the respective antisense sequence. Nevertheless, several challenges remain before effective antisense agents can be identified. One major limitation in the use of antisense PNAs is their restricted uptake into cells. To improve translocation, many cargos, including PNAs, have been coupled to CPPs (29, 30).

In this work, we compared the antimicrobial efficiency of K8-, TAT-, and (RXR)_4_XB-conjugated antisense PNAs in the S. pneumoniae strains TIGR4, D39, and 19F. K8-coupled antisense PNAs did not show bactericidal activity in any of the S. pneumoniae strains tested. Previously, we showed that K8-coupled anti-*gyrA* PNAs affected the growth of S. pyogenes, whereas no effect was mediated by polyarginine-coupled PNAs (22). In eukaryotic cells, polyarginine peptides were more efficient carriers than polylysines (31). R9 coupled to antimicrobial peptides was able to enhance bactericidal activity in Gram-negative bacteria (32). Arginine-rich CPPs use a passive translocation mechanism by inducing membrane multilamellarity and fusion (33). In future experiments, polyarginine should be studied in S. pneumoniae because its surface composition differs from that of S. pyogenes and might support the potency of polyarginine as a CPP.

(RXR)_4_XB-conjugated antisense PNAs reduced bacterial counts of S. pneumoniae TIGR4, D39, and 19F in a killing assay. In all three strains, TAT-antisense PNA constructs were less efficient than (RXR)_4_XB-coupled antisense PNAs. In a previous study, we observed a similar difference between these two CPPs in S. pyogenes (22). Accordingly, (RXR)_4_XB-coupled anti-*rpoA* PNA caused the greatest reduction in CFU/mL in L. monocytogenes, followed by TAT-anti-*rpoA* PNA (20). Overall, (RXR)_4_XB seems to be a potent CPP in Gram-positive bacteria.

CPP-mediated toxicity might challenge the application of carrier peptide-coupled PNAs as antimicrobial agents. In this work, we did not observe (RXR)_4_XB-derived antibacterial effects in S. pneumoniae strains TIGR4 and D39. In contrast, TAT-scPNA reduced the number of S. pneumoniae strain TIGR4 slightly over the course of the time-killing experiment. At high concentrations, both TAT-scPNA and (RXR)_4_XB-scPNA reduced CFU of S. pneumoniae 19F in the killing assay. Previously, we observed a slight nonspecific toxic effect of TAT in S. pyogenes at moderate concentrations (22). Together, these data highlight the fact that antimicrobial CPP-antisense PNA efficiency and specificity depend not only on the species but also on the respective strain investigated. S. pneumoniae serotypes are known to express biochemically distinct capsular polysaccharides (34). Capsules are among the bacterial surface barriers which limit CPP-PNA uptake. We hypothesize, therefore, that differences in capsule size and polysaccharide composition may underlay the differential susceptibility toward CPP-PNAs we observed in this study. The development of beneficial CPP-antisense PNA-based therapies requires extended screening for CPP-PNAs which exhibit antibacterial activity on the most prevalent clinical strains.

Typically, essential genes are used as antisense targets for antibacterial agents (35). We compared the efficiency of two different target genes, *gyrA* and *rpoB*. Both genes have been studied as antisense targets in bacteria and encode structures that are recognized by antibiotics (19, 23, 36). In S. pneumoniae, inhibition of *gyrA* expression was more efficient than targeting of *rpoB*. (RXR)_4_XB-anti-*gyrA* PNA had the highest antibacterial efficiency in S. pneumoniae strains TIGR4, D39, and 19F. TAT-anti-*rpoB* PNA did not affect S. pneumoniae TIGR4 and D39. Only a slight antibacterial effect of TAT-anti *rpoB* PNA could be observed in S. pneumoniae 19F. (RXR)_4_XB coupled to anti-*rpoB* PNA showed bactericidal activity comparable to that of (RXR)_4_XB-anti*-gyrA* PNAs in strains D39 and 19F. We examined the target gene specificity of CPP-PNA treatment in S. pneumoniae TIGR4 by monitoring target gene transcript abundance in the presence of sublethal CPP-PNA concentrations. Under these conditions, moderate target gene mRNA reduction was observed regardless of the CPP-antisense PNA construct used. In conclusion, all four constructs were taken up by the bacterial cell and were able to bind to their respective targets. We hypothesize that distinct translocation efficacy, binding affinity, and other, as-yet unknown factors are responsible for the differences in dose-dependency and kill-kinetics between CPP-antisense PNAs present at lethal concentrations.

In L. monocytogenes and S. aureus, downregulation of *rpoA* translation caused reduced mRNA synthesis and thereby affected downstream genes, including virulence factor genes (20, 23). Similarly, we observed reduced expression of *ply* and *pspA* following treatment with anti-*rpoB* PNAs in S. pneumoniae strain TIGR4. Expression of *ply* was significantly reduced when TIGR4 was incubated with 10 μM TAT-anti-*rpoB* PNA, even though no bactericidal effect of this construct could be observed under these conditions. Knockdown of *rpoB* by antisense PNAs at concentrations which do not result in bacterial clearance will cause reduced virulence factor gene expression, thereby interfering with S. pneumoniae virulence and transmission. Some possible consequences of reduced *ply* expression by S. pneumoniae are decreased inflammation, shedding, and transmission (37).

In this study, (RXR)_4_XB-anti-*rpoB* PNA caused downregulation of virulence factor genes. In a G. mellonella infection model, CPP-anti-*rpoB* PNAs did not increase survival under the conditions tested in this study. However, effects on shedding and transmission are not accounted for in this model. Compared to those in S. pyogenes and L. monocytogenes, the effective concentrations of TAT- and (RXR)_4_XB-antisense PNAs were rather high in S. pneumoniae (20, 22). For instance, MIC values for all constructs were ≥62.5 μM (data not shown) compared to 15.6 μM for TAT-anti-*gyrA* PNA in S. pyogenes (22). Another strategy to improve antibacterial effects of antisense PNAs is a combination therapy with conventional antibiotics. Previous studies have demonstrated that synergistic effects support the bactericidal activity of peptide-coupled antisense PNAs in bacteria (22, 36).

In future studies, more potential targets may be investigated. In uropathogenic Escherichia coli, 11 essential genes with varying expression levels were investigated as potential targets for peptide-conjugated PNAs (38). Three promising target mRNAs were identified for effective growth inhibition: *dnaB*, *ftsZ*, and *rpsH*. This analysis also showed that transcript abundance does not predict target vulnerability. Furthermore, the authors demonstrated that 9mer PNAs were generally as effective in inhibiting bacterial growth as their 10mer counterparts (38).

In addition to knockdown of essential genes, knockdown of virulence genes is becoming increasingly popular (39). Indirect knockdown by limited RNA polymerase production, but also direct antisense knockdown of *ply*, *pspA*, or other well-characterized virulence factors of S. pneumoniae, are potential measures to reduce infection-triggered damage. To tackle the rising number of multidrug-resistant invasive pneumococci, antisense targeting of resistance genes could be utilized to suppress bacterial mechanisms of antibiotic resistance. Numerous studies, in several Gram-negative organisms and in S. aureus, have used anti-resistance gene agents to increase antibiotic efficacy (40–43). In a similar approach, resistance genes could be targeted in S. pneumoniae to achieve resensitization of resistant strains (40, 41, 43).

Overall, we demonstrated that CPP-coupled antisense PNAs could affect the viability of three relevant S. pneumoniae serotypes. The effect was target gene-specific and could be verified *in vivo* using an insect infection model. In future studies, more potential carrier molecules and more target genes should be studied with the aim of identifying constructs with efficient antimicrobial activity toward S. pneumoniae. Promising peptide-coupled antisense PNAs could then be further investigated in murine infection models.

## MATERIALS AND METHODS

### PNA synthesis.

CPP-PNAs were synthesized and purified by high-pressure liquid chromatography (Peps4LS GmbH, Heidelberg, Germany) (44). The sequences of all CPP-PNAs used in this study are listed in Table 1.

### Bacterial strains and culture conditions.

S. pneumoniae strains were grown on Columbia blood agar plates (Becton Dickinson GmbH, Heidelberg, Germany) and cultivated to deceleration growth phase for a maximum of 10 h in brain heart infusion broth (BHI; Oxoid, Wesel, Germany) at 37°C under a 5% CO_2_ atmosphere. Viability was verified by CFU/mL determination. We used serotype 2 strain D39 (NCTC 7466), serotype 19F (RKI 704), and serotype 4 strain TIGR4 (45).

### Bacterial killing assay.

After culturing S. pneumoniae strains in BHI, D39 and 19F were diluted to approximately 10^5^ CFU/mL in phosphate-buffered saline (PBS):BHI (93%:7%), while TIGR4 was diluted to approximately 10^5^ CFU/mL in PBS:BHI (96%:4%). Next, 450-μL volumes of the respective bacterial suspensions, containing approximately 0.5× 10^5^ CFU, were transferred to 2-mL reaction tubes. A 50-μL volume of PNA solution was added to a final PNA concentration of 1 to 20 μM or as indicated. Fifty μL H_2_O served as a mock control. The reaction tubes were incubated for 6 h at 37°C and 7 rpm (Rotor SB3; Stuart, Staffordshire, United Kingdom). The concentration of CPP-PNA that reduced CFU/mL by 50% after 6 h of incubation compared to an untreated control (IC_50_) was calculated by nonlinear regression analysis using GraphPad Prism software (GraphPad Software, San Diego, CA).

For monitoring of killing kinetics, samples were collected 2 to 12 h post-treatment. Viable cell counts were determined by plating appropriate dilutions on BHI agar plates. CFU were determined by visual inspection following overnight incubation at 37°C under a 5% CO_2_ atmosphere. At time point 0, the viable cell count corresponded to 1 × 10^5^ to 3 × 10^5^ CFU/mL. Experiments were performed in at least three independent biological replicates, as indicated in the figure legends.

### Extraction of total RNA.

For RNA isolation, 450 μL bacterial suspension (10^5^ CFU/mL in BHI) was prepared for each experimental condition, treated with CPP-PNA conjugates as indicated, and incubated in a 2-mL reaction tube for 6 h at 37°C and 7 rpm (Rotor SB3; Stuart, Staffordshire, United Kingdom). Subsequently, five samples per condition were pooled. Bacteria were pelleted immediately, shock-frozen in liquid nitrogen, and stored at −80°C until use. Total RNA was extracted according to the protocol published by Li-Korotky et al. (46). Briefly, 30 μg polyinosinic acid (Poly I) was added to each bacterial pellet. TRIzol reagent was added to the bacteria/Poly I mix, which was subsequently disrupted in a homogenizer (Peqlab Biotechnologie GmbH, Erlangen, Germany). RNA was extracted twice with 200 μL chloroform (Thermo Fisher Scientific, Darmstadt, Germany). Following the addition of 5 μL glycogen and 500 μL isopropanol, RNA was precipitated at 13,000 rcf (relative centrifugal force) at 4°C for 15 min. Pellets were suspended in H_2_O. RNA was subsequently treated with acid phenol:chloroform:isoamyl alcohol (125:24:1) (pH 4.5) (Thermo Fisher Scientific) and TURBO DNase (Thermo Fisher Scientific) according to the manufacturer’s instructions. RNA was stored at −80°C until further use.

### Reverse transcription-quantitative PCR.

cDNA synthesis was performed using the Superscript First-Strand Synthesis system for RT-qPCR (Invitrogen, Thermo Fisher Scientific, Darmstadt, Germany). Quantitative PCR amplification was conducted with SYBR Green (Thermo Fisher Scientific) using the LightCycler 480 real-time PCR system (Roche Diagnostics GmbH, Mannheim, Germany). The 5S rRNA gene served as internal control. Relative expression was calculated using the 2^–ΔΔ^*^CT^* method (27). Primers were designed based on the full genome sequence of S. pneumoniae strain TIGR4 (NCBI accession no. NZ_AKVY0100000). Primers are listed as follows: *gyrA*-specific, 5′-CTGAGTATGACCTCTTGGC-3′ and 5′-CCAATCAACTCTGTACGGC-3′; *rpoB*-specific, 5′-GGACCATACTCAACTGTTACCC-3′ and 5′-TCCATTCCTTCATCCAAGTCGC-3′; *ply*-specific, 5′-TTACGCACTAGTGGCAAATCGG-3′ and 5′-CTTTACAGCAGATCATCCAGGC-3′; *pspA*-specific, 5′-TTCCTGAACCAAATGCGTTGGC-3′ and 5′-GTCTTACCTTCAGGATCAAGGC-3′; 5S-specific, 5′-CGATAGCCTAGGAGATACACC-3′ and 5′-GGGCTTAACTTCTGTGTTCGG-3′.

### Cell based infection model.

The human pharynx carcinoma epithelial cell line Detroit 562 (ATCC CCL138) was cultivated in Dulbecco’s modified Eagle medium (DMEM)/F12 (1:1) (Thermo Fisher Scientific, Schwerte, Germany) supplemented with 10% fetal bovine serum at 37°C and 5% CO_2_. Confluent Detroit 562 cells were infected with S. pneumoniae TIGR4 at an MOI of 5. Cultures were treated with 20 μM CPP-PNAs or 1 μg levofloxacin, respectively, and incubated for 3 h at 37°C, 5% CO_2_. After incubation, the supernatant was collected. Adherent cells were detached with Accutase solution (Merck KGaA, Darmstadt, Germany), collected by centrifugation for 10 min at 4,000 × *g*, and combined with the supernatant. Cells were pelleted by centrifugation, washed twice with PBS, and suspended in PBS. CFU/well was determined following serial dilution and plating on Todd Hewitt Broth, supplemented with Yeast, (THY) agar plates.

### Cytotoxicity assays.

Confluent Detroit 562 cells were treated with 20 μM CPP-PNAs or 1 μg levofloxacin, respectively, and incubated for 3 h at 37°C, 5% CO_2_. Crystal violet staining was performed to determine biomass. Cells were washed with PBS and fixed with 100 μL methanol for 10 min. Subsequently, cells were washed with PBS containing 0.005% Tween 20, incubated with 0.1% crystal violet solution for 10 min, and washed with H_2_O. The remaining crystal violet was solved in 100 μL 33% acetic acid for 10 min. Next 70 μL solution was transferred into fresh wells. Optical density was measured at 600 nm. LDH leakage by Detroit 562 cells after treatment with 20 μM CPP-PNAs or 1 μg levofloxacin was determined using the CyQUANT LDH Cytotoxicity Assay kit (Thermo Fisher Scientific, Darmstadt, Germany) according to the manufacturer’s instructions.

### *G. mellonella* infection model.

Larvae of the greater wax moth (G. mellonella) were obtained from Bugs-International GmbH (Irsingen/Unterfeld, Germany). For infection experiments, S. pneumoniae TIGR4 was grown for 7 h in BHI, washed twice in a 0.9% NaCl solution, and suspended in 0.9% NaCl to a final concentration of approximately 1 × 10^8^ CFU/mL. Larvae weighing 150 to 200 mg were infected with 1 × 10^6^ to 3 × 10^6^ CFU/larva. Bacteria were injected into the hemocoel of the larvae between the last two pairs of prolegs using a microapplicator (World Precision Instruments, LLC, Sarasota, FL) and a fine-dosage syringe (Omnican-F, 0.01 to 1 mL, 0.30 × 12 mm; B. Braun AG, Melsungen, Germany). As mock control, 0.9% NaCl (PNA experiment) or 0.9% NaCl/3% DMSO (levofloxacin experiment) was injected. For CPP-PNA treatment, larvae were injected 30 min postinfection with 10 nmol CPP-PNA/larva. For levofloxacin treatment, larvae were injected 30 min postinfection with 1 or 10 μg levofloxacin/larva. Larvae were incubated for 7 days, and survival was monitored daily (22).

For determination of bacterial load, larvae were infected and subsequently treated with NaCl (mock), CPP-PNAs, or levofloxacin (positive control). At 24 h after infection, larvae were homogenized. Following serial dilution, the suspension was plated on selective medium (BD Columbia CNA Agar with 5% sheep blood; Becton Dickinson GmbH, Heidelberg, Germany). Agar plates were incubated overnight at 37°C, 5% CO_2_. CFU/larva was determined by visual inspection. Colony identity was verified on a random basis by colony PCR using *ply*-specific primers.

### Statistical analyses.

All experiments were performed at least three times or as indicated in the respective figure legends by sample size (*n*). Statistical significance was determined using the tests indicated in the respective figure legends. Statistical analyses were performed using GraphPad Prism 7 software.

## Supplementary Material

Reviewer comments
