# Supplementary material for: Antimicrobial Activity of Peptide-Coupled Antisense Peptide Nucleic Acids in Streptococcus pneumoniae
Source: Microbiol Spectr. 2022 Nov 2;10(6):e00497-22. doi: 10.1128/spectrum.00497-22 (PMC9784828; doi:10.1128/spectrum.00497-22)
Supplement: Supplemental file 1 — Supplemental material. Download spectrum.00497-22-s0001.pdf, PDF file, 0.4 MB [file spectrum.00497-22-s0001.pdf]

Supplemental Material

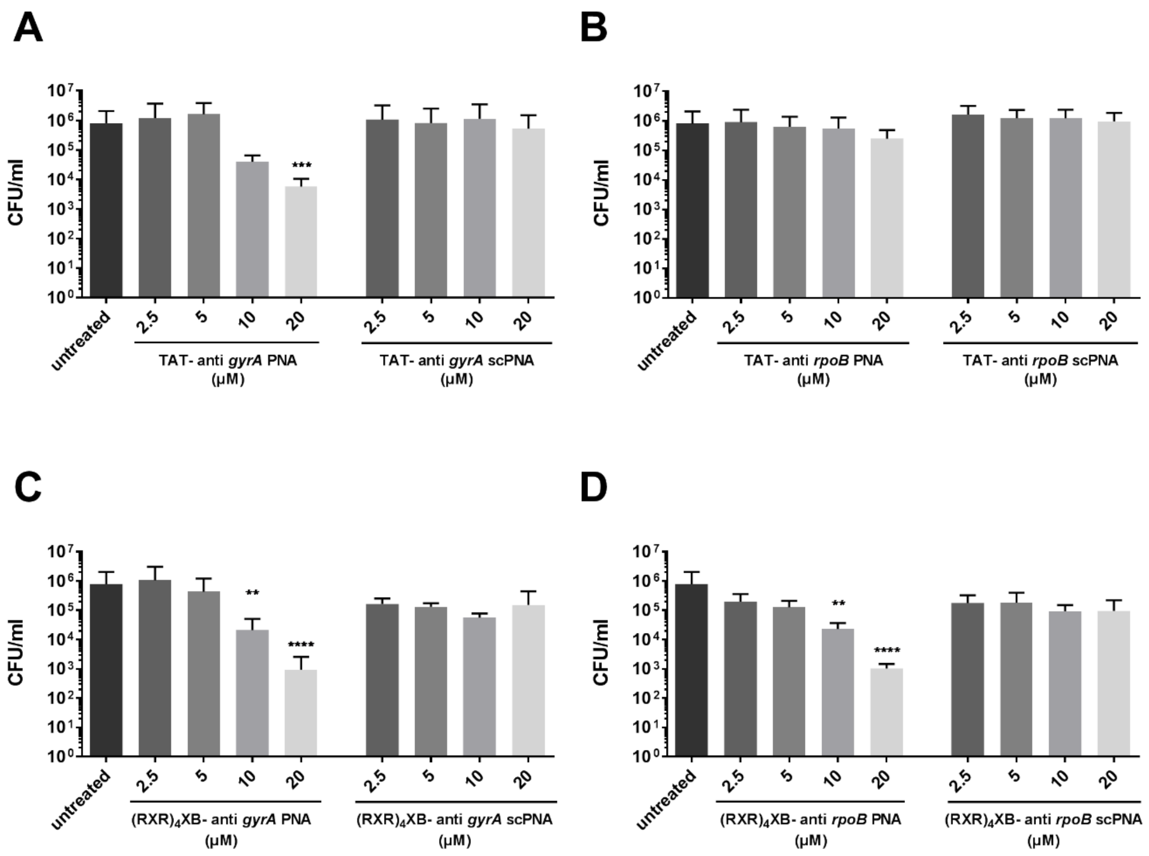

Suppl. Figure 1: Concentration-dependent reduction of the pneumococcal CFU/ml following treatment of *S. pneumoniae* D39 with CPP-antisense PNAs for 6 h. Scrambled PNA controls are indicated as scPNA. A: Treatment with TAT-anti-*gyrA* PNAs. B: Treatment with TAT-anti-*rpoB* PNAs. C: Treatment with $(RXR)_4$XB-anti-gyrA PNAs. D: Treatment with $(RXR)_4$XB-anti-*rpoB* PNAs. Data are presented as mean values and standard deviation. Statistical significance was determined by one-way ANOVA, multiple comparisons. Differences between PNA conjugate samples and mock control (untreated) were expressed as: $p \leq 0.01$ (**); $p \leq 0.001$ (***); $p \leq 0.0001$ (****). Sample size: n = 5.

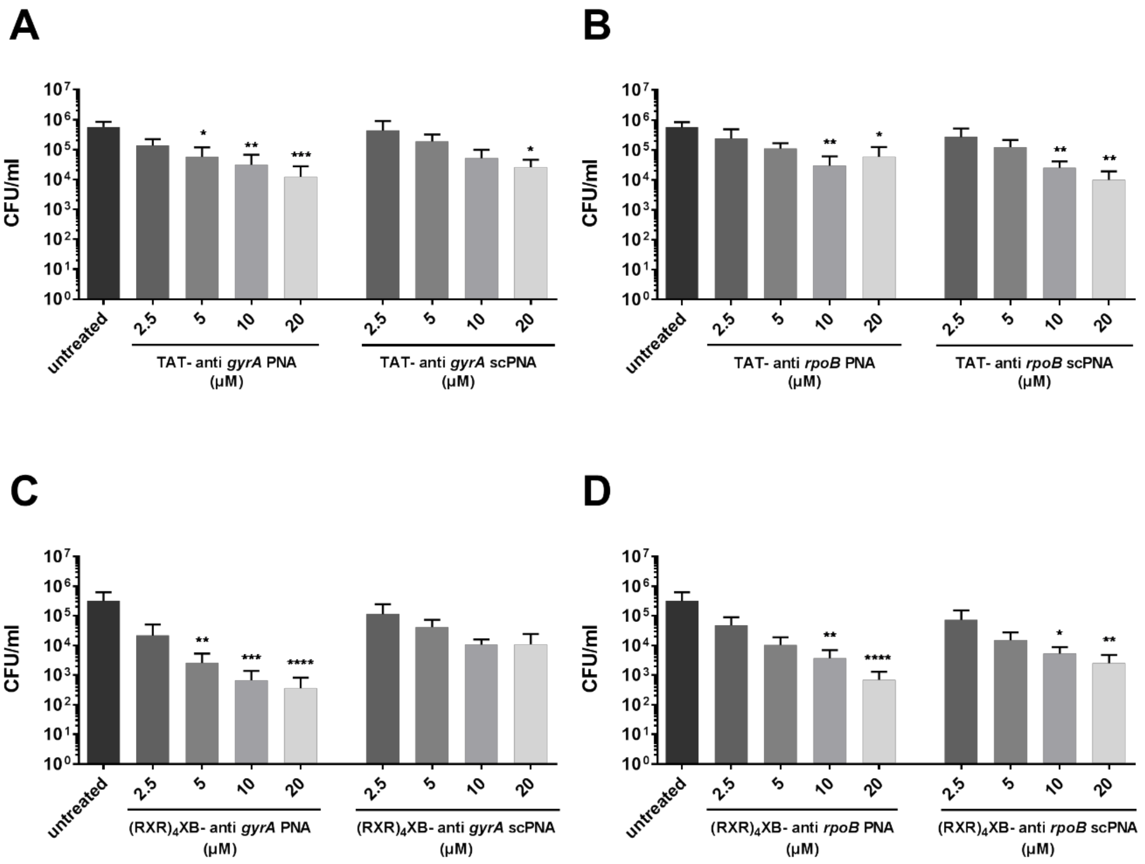

Suppl. Figure 2: Concentration-dependent reduction of the pneumococcal CFU/ml following treatment of *S. pneumoniae* 19F with CPP-antisense PNAs for 6 h. Scrambled PNA controls are indicated as scPNA. A: Treatment with TAT-anti-*gyrA* PNAs. B: Treatment with TAT-anti-*rpoB* PNAs. C: Treatment with $(RXR)_4$XB-anti-gyrA PNAs. D: Treatment with $(RXR)_4$XB-anti-*rpoB* PNAs. Data are presented as mean values and standard deviation. Statistical significance was determined by one-way ANOVA, multiple comparisons. Differences between PNA conjugate samples and mock control (untreated) were expressed as: $p \leq 0.05$ (*); $p \leq 0.01$ (**); $p \leq 0.001$ (***); $p \leq 0.0001$ (****). Sample size: n = 5.

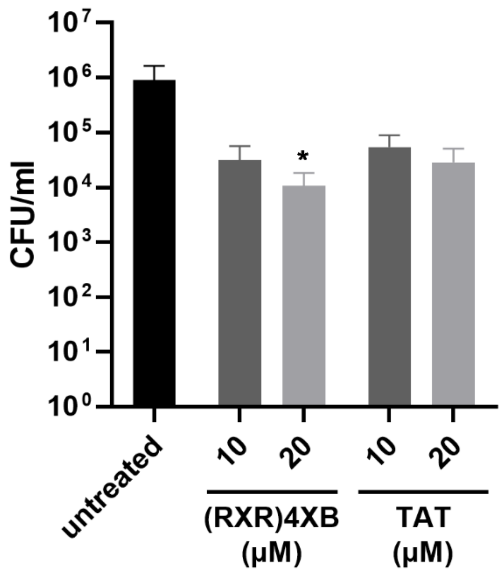

Suppl. Figure 3: Reduction of pneumococcal CFU/ml following treatment of *S. pneumoniae* TIGR4 with CPPs for 6 h. Data are presented as mean values and standard deviation. Statistical significance was determined by one-way ANOVA, multiple comparisons. Differences between CPP-treated samples and mock control (untreated) were expressed as: $p \leq 0.05$ (*). Sample size: n = 3.

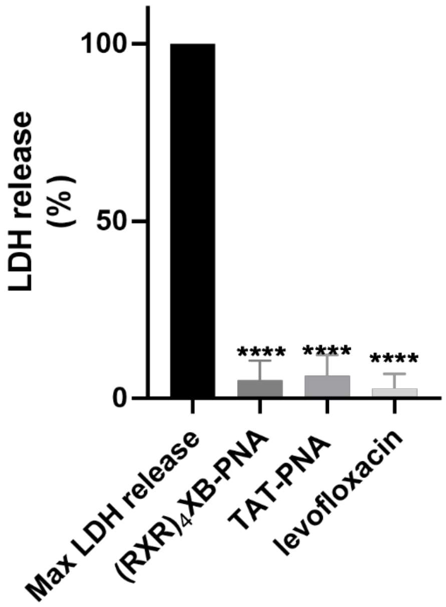

Suppl. Figure 4: LDH release following treatment of Detroit 562 cells with CPPs and levofloxacin. Detroit 562 cells were treated with 20 µM CPPs or 1 µM levofloxacin, respectively. Data are presented as mean values and standard deviation. Statistical significance was determined by one-way ANOVA, multiple comparisons. Differences between LDH release of treated samples and maximum LDH release were expressed as: $p \leq 0.0001$ (****). Sample size: $n = 4$.
